# Depletion of β1,6-*N*-acetylglucosaminyltransferase reduces E-selectin binding capacity and migratory potential of human gastrointestinal adenocarcinoma cells

**DOI:** 10.1016/j.neo.2024.101083

**Published:** 2024-11-14

**Authors:** Lisa Staffeldt, Hanna Maar, Julia Beimdiek, Samuel Chambers, Kristoffer Riecken, Mark von Itzstein, Falk F.R. Buettner, Arun Everest-Dass, Tobias Lange

**Affiliations:** a Institute of Anatomy and Experimental Morphology, University Cancer Center Hamburg, University Medical Center Hamburg-Eppendorf, 20241, Hamburg, Germany; b Institute of Anatomy I, Jena University Hospital, 07743, Jena, Germany; c Comprehensive Cancer Center Central Germany (CCCG); d Institute of Clinical Biochemistry, Hannover Medical School, 30625, Hannover, Germany; e Proteomics, Institute of Theoretical Medicine, Faculty of Medicine, University of Augsburg, Augsburg, Germany; f Institute for Glycomics, Griffith University, Gold Coast Campus, Gold Coast, QLD4222, Australia; g Research Department Cell and Gene Therapy, Department of Stem Cell Transplantation, University Medical Center Hamburg-Eppendorf, 20246, Hamburg

**Keywords:** β1,6-*N*-acetylglucosaminyltransferase, GCNT3, Gastrointestinal adenocarcinoma, E-selectin, Sialyl-Lewis A, Tumor cell migration

## Abstract

The commonly altered glycosylation of tumor cells is a hallmark of tumor progression and metastasis formation. One prominent example is the interaction of sialylated glycans at the tumor cell surface with endothelial (E)-selectin as an early event of an adhesion cascade that enables extravasation of circulating tumor cells (CTCs) into distant tissues. In a previous study, we identified GCNT3 (mucin-type core2/ core4 β1,6-*N*-acetylglucosaminyltransferase) highly over-expressed in gastrointestinal adenocarcinoma cells that facilitate the canonical E-selectin ligands sialyl-Lewis A and X (sLeA/X) for E-selectin binding and endothelial adhesion. Here we show that shRNA-mediated, stable depletion of GCNT3 reduced sLeA (tumor marker CA19-9) presentation on two out of three tested human gastrointestinal adenocarcinoma cell lines, concurrently showing reduced static E-selectin binding. Significant effects of GCNT3 depletion on dynamic, shear-resistant tumor cell adhesion on immobilized E-selectin as well as endothelial cells were only partially and inconsistently observable as were effects on tumor cell proliferation (2D) or 3D colony formation. Nevertheless, tumor cell migration was consistently reduced upon GCNT3 depletion in all tested cell lines. Detailed glycome analyses revealed that GCNT3 depletion caused cell line-specific alterations in *N*- and *O*-glycans as well as glycosphingolipids, collectively mainly associating with decreased Core-2 structures resulting in varied abundance of sialylation and Lewis antigen with consistent phenotypic changes. Distinctive *N*- and *O*-glycosylation features were found to be inherent to specific cell types. These findings suggest GCNT3 products as possible carriers of sLeA and static E-selectin binding sites as well as common pro-migratory glycans in human gastrointestinal cancer.

## Highlights

• CA19-9 expression depends on GCNT3 expression in some GI cancers.
• E-selectin binding by tumor cells is commonly reduced upon GCNT3 depletion.
• tumor cell migration is diminished by GCNT3 depletion in GI cancers.
• GCNT3 depletion affects the *N*- and *O*-glycome and glycolipidome.

## Introduction

Metastatic tumor cells engage in a variety of cell-cell and cell-matrix interactions as they circulate through the body under rapidly changing environmental conditions. Many if not all of these interactions are initiated through the outermost cell layer - the glycocalyx - which contains glycoproteins, glycolipids and proteoglycans. Over the past decades, a multitude of studies has started to decipher the functional roles of distinct sugar residues or classes of glycans during tumor progression and metastasis (reviewed in e.g. Mereiter et al. [1]). Briefly, the most well-known alterations of glycosylation in metastasis mainly comprise altered *N*-glycan branching and fucosylation, mucin expression and truncated *O*-glycans, sialic acid expression, altered glycosphingolipids, and changes in hyaluronan and sulfated glycosaminoglycans [2].

In particular, it has long been recognized that the metastatic phenotype of tumor cells is regulated by sialylated glycans. In short, sialylation is known to affect galectin binding (α2,6- vs. α2,3-sialylation), integrin function (*e.g.* α2,6-sialylation of *N*-glycans on β1 integrin), Fas-mediated apoptosis (α2,6-sialylation of the Fas receptor), invasion (sialyl-Tn antigen), and extravasation [3]. The latter step is mediated by two structural isomers of a tetrasaccharide with the common structure Siaα2,3Gal(Fucα1,4)GlcNAc, but differing in the linkage of Gal to GlcNAc which can be either β1,3 (sialyl-Lewis A, sLeA) or β1,4 (sialyl-Lewis X, sLeX). Both structures are elevated in cancer and function as ligands for selectins, thereby facilitating adhesion of tumor cells to the endothelium of the vascular wall at a distant site and subsequent extravasation [2]. *In vivo* studies using spontaneous metastasis mouse models of human colon, breast and lung cancer demonstrated a remarkable loss of metastasis counts in selectin-deficient mice [[4], [5], [6]]. In close analogy, selectins mediate the adhesion of pancreatic cancer cells from the peritoneal fluid to the mesothelial lining of the abdominal cavity during peritoneal carcinosis [7].

We could previously show that human tumor cells from various entities differ remarkably in their carbohydrate ligands for E-selectin, which determines distinct endothelial adhesion patterns ^8^: tumor cell lines of non-epithelial origin (melanoma, osteosarcoma, small cell lung cancer) commonly lack expression of the canonical E-selectin ligands sLeA/X; nevertheless, they adhere to endothelium in an E-selectin-dependent manner, although with low E-selectin binding affinity. Such tumor cells can interact with isolated, immobilized rhE-selectin under shear stress conditions (laminar flow), but not during static incubation. Pharmacologic reduction of E-selectin expression by approximately 60 % (using the clinically approved proteasome inhibitor bortezomib) is in case of these tumor cell lines sufficient to impair endothelial adhesion *in vitro* and spontaneous lung metastasis *in vivo*. In sharp contrast, tumor cells with detectable sLeA/X expression, such as gastrointestinal (GI) adenocarcinomas, bind E-selectin with high affinity, even in the absence of shear stress; in case of these tumors, the bortezomib -mediated reduction in E-selectin was neither sufficient to impair endothelial adhesion *in vitro* nor to reduce spontaneous metastasis *in vivo* [8]. In the same study, we profiled glycosyltransferase expression levels in the different tumor cell subsets and discovered a remarkable up-regulation of the gene *GCNT3*, encoding a mucin-type core 2/ core 4 β1,6-*N*-acetylglucosaminyltransferase (GCNT3), in the sLeA/X-positive GI adenocarcinoma cells. This finding suggested GCNT3 as a key enzyme in the synthesis of high-affinity E-selectin ligands sLeA/X.

GCNT3 produces core 2 and core 4 *O*-glycan structures and is hence crucially involved in mucin-type biosynthesis [9]. Interestingly, GCNT3 appears to play divergent roles in tumor progression depending on the tumor type: in colorectal cancer, *GCNT3* as well as GCNT3 levels are down-regulated, low levels are linked to higher risk of relapse, and its re-expression causes growth inhibition [9,10]. In hepatocellular carcinoma, *GCNT3* is also downregulated in metastatic compared to non-metastatic clinical cancer specimens [11]. In contrast, high GCNT3 levels predict poor outcome in pancreatic cancer and the mucinous products of this enzyme are associated with aggressive tumorigenesis [12].

Here, we investigated the functional consequences of *GCNT3* depletion in human GI (colorectal, pancreatic and gastric) adenocarcinoma cells, specifically focusing on sLeA/X expression, endothelial adhesion and E-selectin binding. In addition, we considered the effects of *GCNT3* depletion on tumor cell proliferation, colony-forming capacity and migration. Furthermore, we analyzed the effects of *GCNT3* depletion on the tumor cell glycome. Utilizing porous graphitized carbon liquid chromatography (PGC-LC)-MS/MS in negative ion mode, we characterised and quantified the *N*- and *O*-linked protein glycome. This method facilitates an effective separation of isomeric species and allows for a detailed structural analysis of the glycan antigens. Similarly, we utilized capillary gel electrophoresis coupled to laser-induced fluorescence detection (xCGE-LIF) to determine the lipid linked glycosylation.

## Materials and methods

### Cell culture

Human colorectal cancer cells HT29 (ECACC, Porton Down, UK), gastric cancer cells GC5023[13], and pancreatic cancer cells PaCa5061 [14] were cultivated at 37°C in H_2_O-saturated atmosphere with 5 % CO_2_. HT29 and GC5023 cells were grown in RPMI-1640 supplemented with 10 % fetal calf serum (FCS) and 1 % penicillin (50 U/ml)/ streptomycin (50 µg/ml) (all from Thermo Fisher Scientific). PaCa5061 cells were grown in RPMI-1640 GlutaMAX + 10 % FCS and 1 % P/S. All functional assays comparing control (neg) vs. GCNT3 knockdown (kd) cells were carried out at similar passage.

Primary human umbilical vein endothelial cells (HUVECs) were from PromoCell (Heidelberg, Germany) and cultured in endothelial cell medium supplemented with 5 % FCS, 1 % endothelial cell growth supplement (ECGS, PromoCell), and 1 % P/S under standard conditions. HUVECs were used in functional assays until passage six only.

Conditioned media (CM) were generated by harvesting serum-containing medium from 70-80 % confluent PaCa5061 cell cultures in T75 or T175 culture flasks. Collected media were filtered through a sterile filter with a pore size of 0.22 µm (Millex-GP Syringe Filter Unit, Merck Millipore). The CM was freshly prepared before each application and not stored.

### Lentiviral transduction of GCNT3-targeting shRNA

Control (neg) and GCNT3 kd derivatives of the HT29 cell line were established by lentiviral transduction within the framework of a previous study [8]. For the present study, the same protocol was used to generate control (neg) and GCNT3 kd derivatives of GC5023 and PaCa5061 cells. As in the study before, the Mission shRNA assortment (Sigma, GCNT3 kd: TRCN0000279664, neg: SHC002) and the pLKO.1-puro vector were used. The transduced tumor cells were selected by adding 1 µg/ml puromycin to the culture media for two weeks. Afterwards, the puromycin concentration was reduced to 0.5 µg/ml and kept in the culture media for the entire experiment. Transduction efficiency was quantified by WB at several passages.

### Western blot analysis

Whole cell protein extracts were generated using RIPA buffer as previously described [8]. Protein concentrations were determined using the Pierce BCA Protein Assay Kit (Thermo Scientific) in accordance with the manufacturer's instructions. Differences in GCNT3 levels in parental, control transfected (neg) and GCNT3 kd cell extracts were determined by WB as described [15]. For protein detection, the following primary antibodies were used: mouse monoclonal anti-GCNT3 (D-7, Santa Cruz, Dallas, TX, USA), diluted 1:200; mouse monoclonal anti-GAPDH (Proteintech, Manchester, UK), diluted 1:250. Detection was done using SuperSignal West Pico PLUS Chemiluminescent Substrate (Thermo Scientific) and the imaging system FUSION Solo S (VILBER).

### Flow cytometry

Flow cytometric determination of sLeA/X levels and static rhE-selectin binding capacity was carried out as described before [8,13,16].

### Laminar flow adhesion assays

Quantifications of dynamic adhesive events of human tumor cells on immobilized rhE-selectin and HUVEC monolayers under laminar flow conditions (distinguished into firm, rolling, and tethering adhesion) were performed as previously published [8,13,16].

### Colony formation assay

To enable colony formation in a 3D environment, tumor cells were seeded in a gel-like soft agar matrix (2-Hydroxyethylagarose). The required amount of cell culture media and CM (the latter required for PaCa5061 cells only as they do not grow well at low seeding density in 3D) were pre-warmed to 42°C. The solid agarose stock solution was heated in a microwave for 2-3 min to enable liquefaction. To avoid adherent growth of tumor cells, a 24-well plate was pre-coated with 0.8 % agar diluted in culture medium. In case of PaCa5061 cells, the harvested CM was mixed 1:2 with fresh medium and used for diluting agar. 250 µl of the diluted 0.8 % agar were pipetted into each well of a 24-well plate. Afterwards, agar polymerization was allowed by storing the plate for 30 min at 4°C. Next, tumor cells were detached from conventional culture using 0.05 % Trypsin-EDTA (Gibco) and cell suspensions of 600 cells/ ml (HT29 and GC5023 cells) or 1800 cells/ ml (PaCa5061 cells) were prepared in fresh medium (HT29/ GC5023) or fresh medium mixed 1:2 with CM (PaCa5061), in both cases containing 0.3 % agarose. 250 µl of these suspensions were pipetted onto the pre-coated agar into each well. After polymerisation for 30 min at 4°C, 1 ml medium without additives (HT29/ GC5023) or medium mixed 1:2 with CM (PaCa5061) were added per well. Tumor cells were allowed to form colonies in soft agar over a period of 10 days under standard culture conditions and half of the medium (HT29/ GC5023) or medium/ CM mixture (PaCa5061) was changed as appropriate. Afterwards, all colonies per well (n=4 wells (HT29) or n=3 wells (GC5023, PaCa5061)) were photographed at 10x magnification using an Axiovert 35 Inverted Phase Contrast Microscope (Zeiss, Jena, Germany). The numbers and mean areas of colonies per well were analysed using ImageJ 1.53 software (NIH, NY, USA).

### Proliferation assay

To determine tumor cell proliferation, cells taken from 70-80 % confluent culture were seeded in a number of 50,000 or 200,000 cells (HT29/ GC023 or PaCa5061, respectively) per T25 culture flask using culture media with puromycin in a total volume of 5 ml per T25. Cells were incubated under standard culture conditions and counted manually on day 1 to day 4.

### Migration assay

Tumor cell migration assays were carried out using the Platypus system (Platypus Technologies, LLC, Madison, WI, USA). Silicon stoppers were placed with firm contact into the centres of 96-well bottoms. Afterwards, 5 × 10^4^ cells were seeded in each well by pipetting 200 µl of the cell suspension onto each side of the plugs to ensure even distribution of cells. After 24 hours of incubation, the stoppers were removed from the wells, thereby leaving a 2 mm, circular, cell-free area. Subsequent tumor cell migration into the cell-free area was monitored by taking photomicrographs at 2.5x magnification every 24 hours for a total of four days. The remaining cell-free area in µm^2^ at given time points was analysed using ImageJ 1.53c. Subsequently, the percentage of the initially cell-free area that was migrated by tumor cells was calculated.

### Glycosphingolipid analysis

Glycosphingolipid (GSL)-derived glycans were assessed by multiplexed capillary gel electrophoresis coupled to laser-induced fluorescence detection (xCGE-LIF), as previously described by Rossdam *et al* [17]. Briefly, GSL-derived glycans were released by LudgerZyme Ceramide Glycanase (CGase) (Ludger, Oxfordshire, UK) digestion from 5 × 10^6^ cells (2 × 10^6^ in case of PaCa5061), fluorescently labeled with 8-aminopyrene-1,3,6-trisulfonic acid (APTS, Sigma Aldrich), and subjected to xCGE-LIF using a remodeled ABI PRISM 3100-Avant Genetic Analyzer equipped with Run 3100-Avant Data Collection Software v.2.0 (Thermo Fisher Scientific). Resulting data were further analyzed with GeneMapper Software v.3.7. Peak annotation to specific GSL-derived glycan structures was based on manual migration time matching to our in-house established database. Normalized signal intensities were calculated in relation to the internal standard (0.083 ng APTS-labeled Oligomannose 6; Prozyme, Hayward, CA).

### *N*- and *O*-glycan mass spectrometry analysis

The procedure for glycan extraction and the enzymatic release from proteins and lipids underwent minor modifications from established protocols [[18], [19], [20]]. The cell pellets (4 × 10^6^ cells) were lysed using 500 µl of RIPA Buffer (Genesearch; Cat No 9806S), supplemented with Roche complete protease inhibitor cocktail (CO-RO - Roche; Merck - 11697498001) and incubated overnight at 4°C. This was followed by a 10-minute sonication in a sonication bath and centrifugation at 16,000 x g for 10 minutes, after which the supernatant was transferred to a new microfuge tube. A chloroform:methanol:water mixture (1:2:2) was used for phase separation to separate the proteins from lipids and other impurities. After vortexing and chilling for 1 hour, the samples were centrifuged at 10,000 rpm for 10 minutes using a table-top centrifuge. The upper aqueous layer was removed, and proteins were precipitated by adding double the volume of methanol and vortexing. Further centrifugation at 10,000 RPM for 10 minutes allowed for the removal of the top lipid layer, and the protein pellet was resuspended in 100 µl of 4 M urea and 3 % SDS for quantification using the BCA assay.

Protein lysates (20 µg) from each replicate were dot blotted onto ethanol-pre-wetted PVDF membranes, including controls for glycan (20 µg of bovine fetuin). The membranes were left to dry overnight at room temperature, then washed in methanol and water. To confirm and visualize the immobilized protein, the membranes were stained with direct blue staining solution, destained, and placed in water. Protein spots (6 mm diameter) were cut out and soaked in 1 % PVP40 solution in wells, washed, and then treated with PNGase F (1000 U, New England Biolabs) in water overnight at 37°C for *N*-glycan release. The wells were then sonicated, rinsed, and acidified with ammonium acetate, followed by reduction of released *N*-glycans in alkaline conditions (NaBH4 in KOH) and neutralized post-cooling. Sodium removal was achieved using cation exchange microcolumns packed with Dowex 50W X8 in ZipTip C18 tips, followed by vacuum drying in a SpeedVac Concentrator.

Desalting was followed by purification using porous graphitised carbon (PGC) chromatography. PGC columns in ZipTip C18 tips were pre-treated with elution and loading buffers, and dried samples were reconstituted and passed through these columns. Enriched glycans were eluted, dried, and stored at -20°C for MS analysis.

For *O*-glycan release, the proteins on PVDF membranes were treated with high alkaline conditions (NaBH4 in KOH), neutralized, desalted, and purified similar to *N*-glycans. The released glycans were processed similarly to *N*-glycans.

Sample handling and UHPLC injections for MS analysis were conducted using an Ultimate 3000 system, with separation achieved using gradients for *N*- and *O*-linked glycans. Glycans were detected using an amaZon ETD speed ion trap in negative ion mode. The reconstitution of the samples was done in 20 μl of water, followed by an injection of 5 μl into the chromatography system. A Hypercarb column, of dimensions 30 × 1 mm with a 3 μm particle size, maintained at a steady temperature of 50°C was utilized for separation. This was achieved using the solvents buffer A (comprising 10 mM ammonium bicarbonate) at a flow rate of 15 μl per minute buffer B, which contained 80 % acetonitrile (MeCN) in 10 mM ammonium bicarbonate. The separation was conducted by applying a linear gradient that ranged from 0 % to 50 % buffer B over 65 minutes.

The liquid chromatography (LC) system was integrated with an amaZon ETD speed ESI ion trap mass spectrometer (MS) from Bruker. The capillary voltage was set at 3300 V, with a dry gas temperature of 300˚C flowing at 6 l/min and a nebulizer pressure of 0.6 bar. MS spectra were collected across a mass-to-charge ratio (*m/z*) spectrum of 250-1600 in Ultrascan mode, setting the target mass for smart parameter setting to *m/z* 900 for *O*-glycans and 1200 *m/z* for *N*-glycans, ion charge control (ICC) to 70,000, and the maximum acquisition time to 200 milliseconds. MS/MS spectra were obtained through collision-induced dissociation across an m/z range of 100-2500, focusing on the three most abundant precursors with an isolation width of m/z 3. The fragmentation threshold was set at 28 %, with a full fragmentation amplitude as part of the Enhanced Smart Frag option, which varied from 30-120 % in 32 milliseconds and the ICC for was adjusted to 100,000.

Data analysis was performed using Compass Data Analysis 4.2 and Skyline 20.1 for structural assignment and quantitation, respectively. The identification of glycans was carried out by assessing their retention time on PGC, examining known biosynthetic routes, and manually analyzing the fragmentation spectra. This analysis was conducted in accordance with established MS/MS fragmentation pathways for *N*- and *O*-glycans in negative-ion mode [21]. Quantitation was based on MS spectra and retention time, calculating the area under the curve for each glycan isomer.

## Statistics

In most analyses, paired t-tests were used to compare values from neg vs. GCNT3 kd samples of similar cell culture passage. Two-way ANOVA and Bonferroni's multiple comparisons test was used to test significance in the migratory behavior of tumor cells at different time points. Unpaired t-tests were used to analyze differences between samples in the glycome analyses.

## Results

### GCNT3 protein levels increase during cultivation and are stably depleted by shRNA

**Fig. 1:**
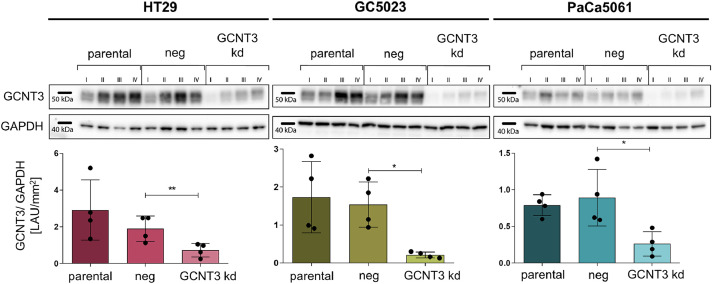
ShRNA-mediated *GCNT3* knockdown in human gastrointestinal adenocarcinoma cells. GCNT3 protein levels relative to loading control (GAPDH) in parental, control transfected (neg) or GCNT3 knockdown (kd) cell line derivatives of three human gastrointestinal adenocarcinoma cell lines as indicated. Note that the GCNT3 protein levels commonly increase during cultivation (passages I-IV). Bars in scatter plots represent mean GCNT3/ GAPDH ratio ± SD from all four passages. LAU = fluorescence intensity unit; *p < 0.05; **p ≤ 0.01 (paired t-test of LAU/ mm² values from protein extracts of corresponding passages).

The three tested human GI adenocarcinoma cell lines HT29 (colorectal cancer), GC5023 (gastric cancer) and PaCa5061 (pancreatic cancer) commonly showed an increase in GCNT3 protein levels in the course of conventional *in vitro* cultivation (time points I to IV in Fig. 1) regardless of whether parental, control transduced (neg) or GCNT3 kd cells were considered (Fig. 1). There was no difference in GCNT3 protein levels between parental and neg cells whereas GCNT3 kd cells showed a significant reduction in GCNT3 (*p*=0.006 in HT29, *p*=0.014 in GC5023, *p*=0.012 in PaCa5061 cells, paired t-test of neg vs. kd samples of similar passage, Fig. 1). GAPDH served as loading control.

### Stable GCNT3 depletion partially affects sLeA/X expression and static E-selectin binding

**Fig. 2:**
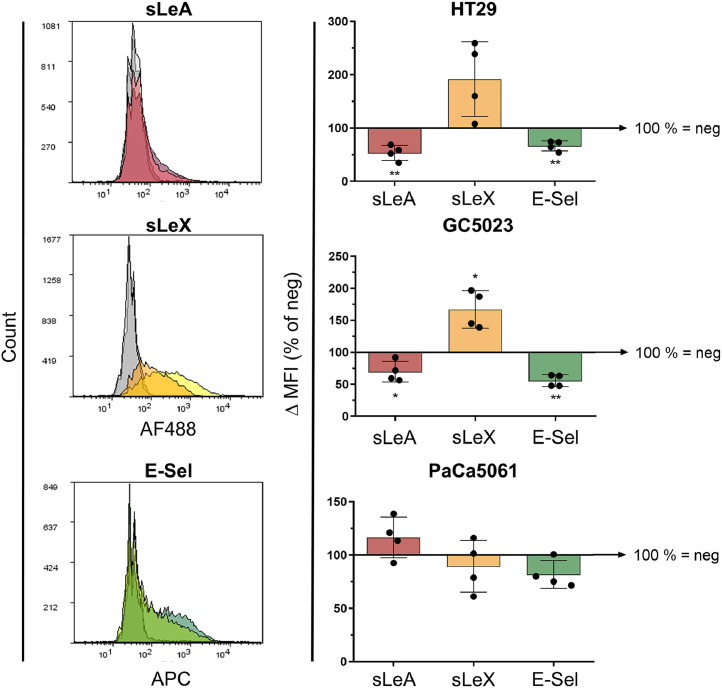
Effects of GCNT3 depletion on sialyl-Lewis antigen levels and static E-selectin binding capacity. Flow cytometric quantification of cell surface sialyl-Lewis A (red) and X levels (yellow) as well as static E-selectin/ IgG1-Fc chimera binding capacity (green) of GCNT3 knockdown cell lines, relative to control (neg = 100 %). The histograms on the left show representative samples of the cell line GC5023 (dark-colored lines indicate neg control and light-colored lines indicate GCNT3 knockdown; grey areas represent binding of antibody isotype or human IgG1-Fc control). Bar charts represent mean ± SD of n = 4 biological replicates. *p < 0.05; **p ≤ 0.01.

The shRNA-mediated reduction in GCNT3 protein levels was accompanied by significantly reduced sLeA cell surface expression in two of the three tested GI adenocarcinoma cell lines (by ∼50 % in HT29 cells, *p*=0.007, and by ∼30 % in GC5023 cells, *p*=0.033, paired t-test of n=4, Fig. 2) which concomitantly showed reduced static rhE-selectin binding (by ∼30 % in HT29 cells, p=0.005, and by ∼45 % in GC5023 cells, p=0.002, paired t-test of n=4, Fig. 2). Interestingly, sLeX expression was concurrently increased on these cells, though in part by trend only (by ∼90 % in HT29 cells, *p*=0.079, and by ∼65 % in GC5023 cells, *p*=0.019, paired t-test of n=4, Fig. 2). In case of PaCa5061 cells, we did not observe any reliable effect of the GCNT3 kd on sLeA/X expression or static E-selectin binding.

### GCNT3 depletion differentially alters dynamic tumor cell adhesion

**Fig. 3:**
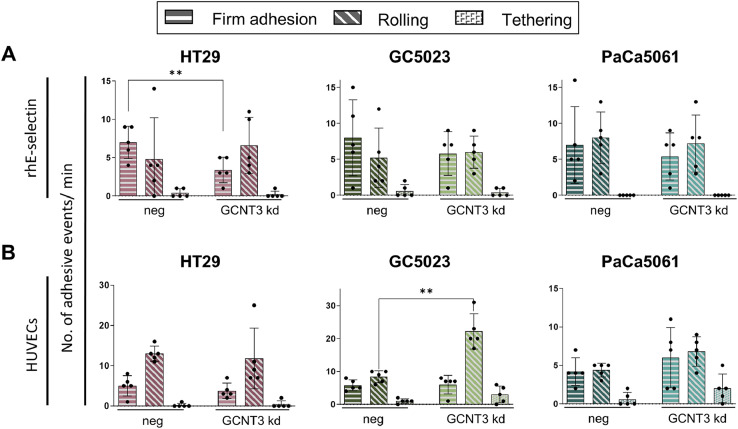
Effect of GCNT3 depletion on shear flow-resistant adhesion of tumor cells. Number of adhesive events per minute of indicated tumor cell line derivatives (distinguished in firm adhesion, rolling and tethering as illustrated) in laminar flow adhesion assays on immobilized rhE-selectin (A) and IL-1a-stimulated human umbilical vein endothelial cells (HUVEC, B). Bar charts represent mean ± SD of n = 5 recordings; **p ≤ 0.01.

Next, we analyzed whether the partial changes in sLeA/X expression and static E-selectin binding upon *GCNT3* kd also lead to notable alterations in the tumor cells’ capability to dynamically bind to E-selectin and to adhere on cytokine-stimulated HUVECs under laminar flow conditions. Of note, we observed a significant reduction in the number of firm adhesions of HT29 cells on immobilized rhE-selectin (by ∼50 %, *p*=0.006, paired t-test of n=5, Fig. 3A). However, laminar flow adhesion assays using HUVECs did not reveal reduced endothelial adhesions (Fig. 3B). The number of rolling adhesions of HT29 cells remained unaffected by the GCNT3 kd in both the dynamic E-selectin binding and laminar flow adhesion assay on HUVECs; tethering adhesions were almost not observable in both assays in case of HT29 cells (Fig. 3). GC5023 cells also mainly showed firm and rolling but largely lacked tethering adhesions on both coatings; while the dynamic binding of these cells to rhE-selectin was not influenced by the kd (Fig. 3A), the number of rolling adhesions on HUVECs sharply increased (by ∼150 %, *p*=0.003, paired t-test of n=5, Fig. 3B). The dynamic E-selectin binding and flow adhesion behavior of PaCa5061 cells on HUVECs, which was also predominantly characterized by firm and rolling adhesions, was not affected by GCNT3 kd (Fig. 3).

### GCNT3 depletion partially affects tumor cell growth in 3D and conventional conditions

**Fig. 4:**
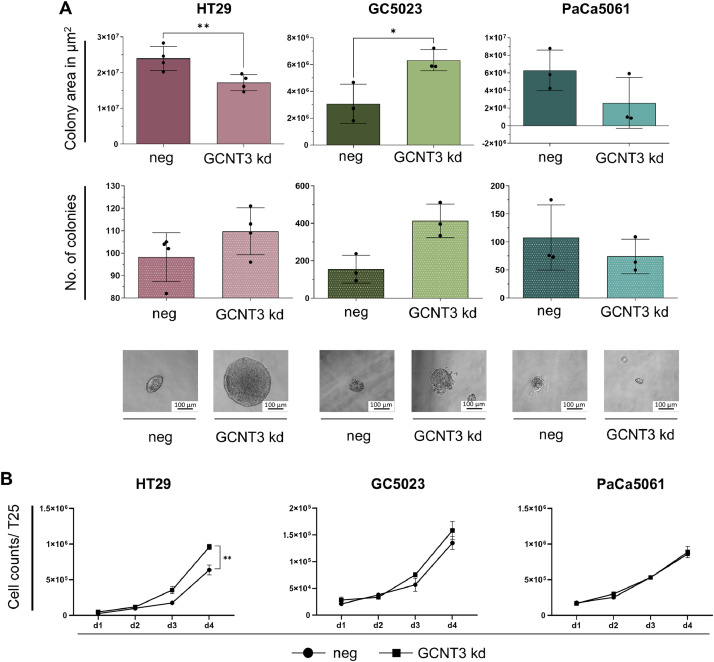
Effects of GCNT3 depletion on colony formation capacity and tumor cell proliferation. Mean total colony area [µm²] (upper row) and mean number of colonies (lower row) per well formed by indicated tumor cell line derivatives in 3D soft agar assays (A). Photomicrographs embedded in (A) illustrate 3D sphere morphology of the counted colonies. Cell counts after seeding 50,000 cells (HT29, GC5023) and 200,000 cells (Paca5061) and cultivation for up to four days (n=3) as indicated under conventional 2D conditions (B). Bar charts represent mean ± SD of n = 4 (HT29 in (A)). *p < 0.05 and **p ≤ 0.01.

Colony formation assays in soft agar revealed that the numbers of established colonies per well were not altered by the *GCNT3* kd in all tested cell lines, although there was a noticeable trend towards more colonies in the kd in case of GC5023 cells (Fig. 4A). Nevertheless, the mean total area of the developed colonies per well was significantly decreased in case of HT29 (by ∼6.5 × 10^6^ µm², *p*=0.0085, paired t-test of n=4, Fig. 4A) and increased in case of GC5023 cells (by ∼3 × 10^6^ µm², *p*=0.017, paired t-test of n=3, Fig. 4A). The colony formation capacity of PaCa5061 cells in soft agar was not altered by GCNT3 depletion.

Under conventional cell culture conditions, the *GCNT3* kd strikingly improved the proliferation of HT29 cells (*p*=0.015, paired t-test of n=3, Fig. 4B). Despite a clearly visible trend to enhanced proliferation also in case of GC5023 cells (*p*=0.053, paired t-test of n=3), we did not observe any further significant effect of the kd on tumor cell proliferation (Fig. 4B).

### GCNT3 depletion impairs migration of gastrointestinal adenocarcinoma cells

**Fig. 5:**
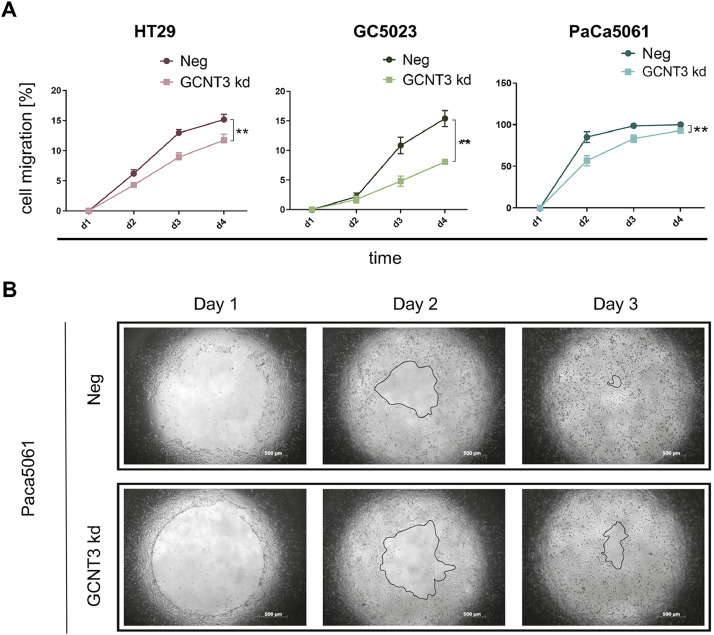
Effect of GCNT3 depletion on tumor cell migration. Cell exclusion zone assay (Platypus assay) was performed to define the migratory potential of indicated tumor cell line derivatives by measuring changes of the cell free area over a period of 4 days. Point-to-line graphs represent two-way ANOVA of mean ± SEM of n = 6 wells at each time point. **p ≤ 0.01.

Testing the migratory behavior of the tumor cells in a platypus assay over a period of four days, we observed that in case of all tested cell lines the GCNT3 kd led to an overall reduction in tumor cell migration (HT29: *p*=0.0069; GC5023: *p*=0.0032; PaCa5061: p=0.0058, two-way ANOVA of n=6 per time point, Fig. 5). The Bonferroni's multiple comparisons tests revealed that significant differences in the migratory capacity occurred on d3 and d4 in case of HT29 and GC5023 cells and on d2 and d3 in case of PaCa5061 cells (Fig. 5).

### Effects of GCNT3 depletion on the tumor cell N- and O-glycoslyation

In the next step, we were interested how the GCNT3 kd affected tumor cell glycosylation. We obtained comprehensive *N*- and *O*-glycan profiles of the three cell lines using our sensitive and robust Porous Graphitic Carbon Liquid Chromatography Electrospray Ionisation Mass Spectrometry (PGC-LC-ESI-MS) [[18], [19], [20]] approach. The three human GI adenocarcinoma cell lines showed similarities in *N*- and *O*-glycans but differences in terms of structural abundance and complexity. The glycan structures were characterized based on their molecular mass, specific fragment ions observed in MS/MS negative ion mode analysis, and their PGC retention times.

**Fig. 6:**
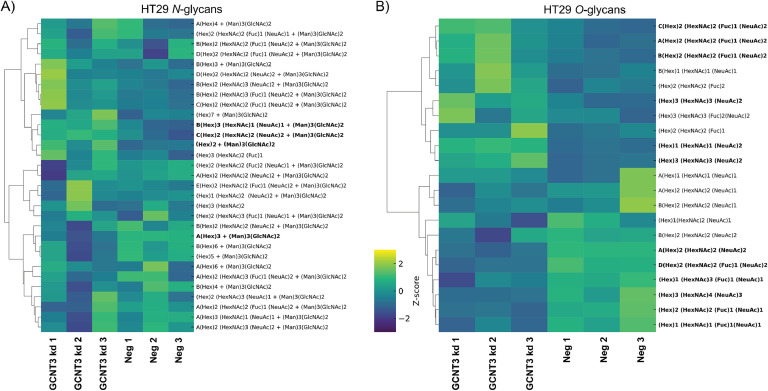
Effects of GCNT3 depletion on *N*- and *O*-glycosylation of HT29 cells. Clustered heatmaps displaying glycan composition profiles as indicated with significantly altered structures (bold) between GCNT3 kd and control (neg) groups (biological triplicates). Unsupervised hierarchical clustering was performed on rows to group glycan compositions with similar expression profiles. Significance was determined by unpaired t-tests and defined by *p* < 0.05.

A total of 31 *N*-glycans were characterized in detail for the HT29 cell line, representing oligomannose, hybrid, paucimannose, bisecting structures, and complex sialylated *N*-glycans as shown in the heatmap in Fig. 6A. Most of the structures were core-fucosylated, except for one terminally fucosylated structure of composition (Hex)2 (HexNAc)2 (Fuc)2 (NeuAc)1 + (Man)3(GlcNAc)2. Oligomannose glycans comprised the highest abundant class with an average relative abundance of 52.6 %. The next most abundant structures were the sialylated structures, amounting to 36.5 % abundance; almost all complex glycans were sialylated. The sialylation contained both α2-3 and α2-6 linked NeuAc sialic acids, as observed by their differential retention time detection on PGC columns [21]. The GCNT3 kd HT29 cells had a similar profile, with only significant differences observed in the abundance of four structures, two oligomannose (Man5 (*p*=0.033) and Man6 (*p*=0.005)) and two sialylated structures: (Hex)2 (HexNAc)3 (Fuc)1 (NeuAc)2 + (Man)3(GlcNAc)2 (*p=*0.033) and (Hex)2 (HexNAc)2 (NeuAc)2 + (Man)3(GlcNAc)2 (*p=*0.016), the former identified as bisected.

The *O*-glycans were characterized similarly to the *N*-glycans. Identification was based on PGC retention time, described biosynthetic pathways, and manual inspection of fragmentation spectra, following known MS/MS fragmentation patterns of *O*-glycans in negative-ion mode. This approach enabled determination of glycan structural features such as core type (1, 2, 3, or 4), I-branch (GlcNAcβ1-6Gal-R), sialylation (α2,3- or α2,6), ABO blood group antigen, presence of Lewis x/a (Galβ1-4/3(Fucα1-3/4)GlcNAc-R), and sialyl-Lewis x/a (NeuAcα2-3Galβ1-4/3(Fucα1-3/4)GlcNAc-R) antigens. Structures with incomplete structural information were categorized as undetermined. Our analysis of the HT29 cell line revealed 21 *O*-glycan structures, consisting of core 1, 2, and 3 structures. We also detected the presence of Lewis and sialyl-Lewis antigens, and the presence of I-branching in one structure (Fig. 6B). The presence of both Lewis x/a (Galβ1-4/3(Fucα1-3/4)GlcNAc-R) antigens is suspected due to the observation of isomeric separation attributed to different elution patterns resulting from the fucose linkages [21]. For example, the precursor ion with *m/z* 1477.53, corresponding to the composition (^A^(Hex)2 (HexNAc)2 (Fuc)1 (NeuAc)2, has four isomers eluting at 13.4, 15.7, 16.1, and 17.6 minutes (Table S1). The described biosynthetic pathway and fragmentation analysis identifies this structure as containing a sialyl-Lewis antigen on the β1-6 GlcNAc arm. The *O*-glycome of HT29 was predominantly sialylated, with the most abundant structure being the core-2 di-sialylated O-glycan (m/z 1331.47; ^A^(Hex)2 (HexNAc)2 (NeuAc)2), accounting for 66.9 % of the total relative abundance across the replicates. The GCNT3 kd HT29 cells’ O-glycan profile showed twelve significantly altered structures, representing an altered glycan phenotype. Most notably, the relative abundance of the most abundant structure, the core-2 di-sialylated *O*-glycan (^A^(Hex)2 (HexNAc)2 (NeuAc)2), dropped by 17.6 %. Meanwhile, the di-sialylated core-1 structure increased to 37 % compared to the control (21.4 %) (*p<0.0001*). The difference in relative abundance of these two structures correlates exceptionally well with GCNT3 kd, resulting in decreased core-2 *O*-glycan. All four isomers carrying the sialyl Lewis antigens described above (*m/z* 1477.53) were found to be significantly reduced (Figures 6B; Table S1). Other *O*-glycans containing Lewis a/x antigens ((Hex)2 (HexNAc)2 (Fuc)1, *p=*0.042); (Hex)2 (HexNAc)2 (Fuc)1, *p=*0.033) were also significantly altered. The only I-branched structure of composition (Hex)3 (HexNAc)4 (NeuAc)3 was also reduced (*p=*0.007) in the GCNT3 kd cells.

**Fig. 7:**
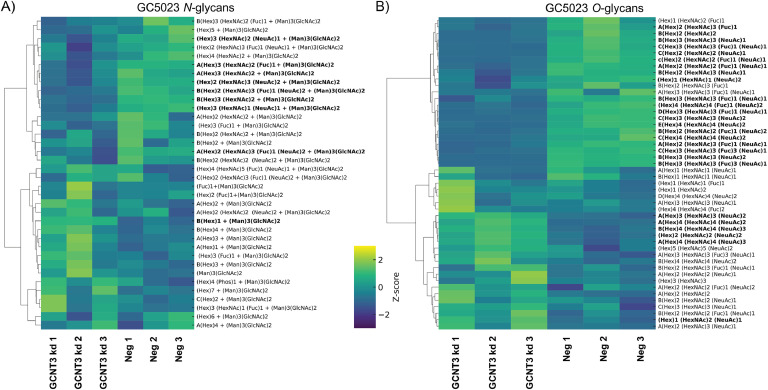
Effects of GCNT3 depletion on *N*- and *O*-glycosylation of GC5023 cells. Clustered heatmaps displaying glycan composition profiles as indicated with significantly altered structures (bold) between GCNT3 kd and control (neg) groups (biological triplicates). Unsupervised hierarchical clustering was performed on rows to group glycan compositions with similar expression profiles. Significance was determined by unpaired t-tests and defined by *p* < 0.05.

The GC5023 cell line exhibited a diverse array of both *N*- and *O*-glycans. A total of 36 *N*-glycan structures were identified (Fig. 7A). Oligomannose structures accounted for 62.7 %, with Man5 being the most abundant, having a relative abundance of 18.34 %. Sialylated *N*-glycans, comprising both α2,3- and α2,6-linked NeuAc, corresponded to 20.8 %, while the remainder of the structures were neutral, hybrid, and paucimannosidic. All observed fucosylation of the *N*-glycans occurred at the core. The GCNT3 kd of GC5023 cells demonstrated nine significant alterations, interestingly. The majority of the structures were featuring the presence of bisecting GlcNAc residues (Table S1), including hybrid bisecting (^A^(Hex)3 (HexNAc)2 + (Man)3(GlcNAc)2, ^B^(Hex)3 (HexNAc)2 + (Man)3(GlcNAc)2) and mono- ((Hex)3 (HexNAc)1 (NeuAc)1 + (Man)3(GlcNAc)2) and di-sialylated bisecting GlcNAc (^A^(Hex)2 (HexNAc)3 (Fuc)1 (NeuAc)2 + (Man)3(GlcNAc)2). The trend in increased bisecting structures in GCNT3 kd GC5023 cells show an indirect effect on GlcNAc-T III, that is encoded by the gene *MGAT3,* on the *N*-glycan biosynthetic pathway.

The *O*-GalNAc-linked glycans of GC5023 cells displayed a wide variety of structures, including core types (1 and 2), I-branching, sialylation (α2,3- or α2,6), blood group antigens, and Lewis as well as sialyl Lewis antigens (Fig. 7B). These comprised 48 unique structures, with some compositions having several isomers. Sialylated structures accounted for 96.8 % of the relative abundance of *O*-glycan species. Interestingly, the GCNT3 kd GC5023 cells did not show a decrease in overall sialylation, maintaining 96.5 %. Of the 48 structures, 28 were found to be significantly altered (Table S1). The changes in *O*-glycan core can be correlated with isomers of (Hex)2 (HexNAc)2 (*p*=0.007), indicating decreased *O*-glycan core-2 branching compared to its linear structure (*p*=0.019) of the same composition in GCNT3 kd GC5023 cells. Also observed were structures with extended LacNAc terminals in the β1-6 GlcNAc arm that are carriers of the sialyl-Lewis antigen (e.g. (Hex)2 (HexNAc)2 (Fuc)1 (NeuAc)2). Interestingly, the isomers that contained Lewis x/a antigens, displayed opposite trends indicating differential regulation of the type 1 or type 2 Lewis antigens.

**Fig. 8:**
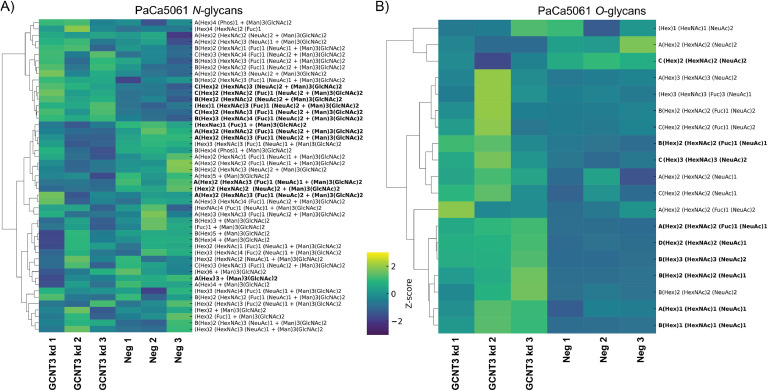
Effects of GCNT3 depletion on *N*- and *O*-glycosylation of PaCa5061 cells. Clustered heatmaps displaying glycan composition profiles as indicated with significantly altered structures (bold) between GCNT3 kd and control (neg) groups (biological triplicates). Unsupervised hierarchical clustering was performed on rows to group glycan compositions with similar expression profiles. Significance was determined by unpaired t-tests and defined by *p* < 0.05.

The *N*-glycans of the PaCa5061 cell line (overall 49 identified) exhibited a distinct profile due to the expression of LacdiNAc (GalNAcβ1-4GlcNAc) terminal motifs (Fig. 8A). In some instances, these LacdiNAc motifs were also sialylated. Here, the most abundant structures were the sialylated species, corresponding to 47.18 %, with oligomannose types amounting to about 42.60 %. The LacdiNAc-containing structures amounted to only 3.27 %. The GCNT3 kd PaCa5061 cells showed an overall increase in sialylation (53.41 %) and a decrease in oligomannose structures (37.36 %). Thirteen of the *N*-glycans were found to be significantly altered, most of which were sialylated *N*-glycans and a decreased Man6 structure (Table S1).

The *O*-glycans of the PaCa5061 cell line exhibited core-1 and -2 structures, with Lewis fucosylation and sialyl-Lewis fucosylation. All detected structures (a total of 19) were sialylated, with both α2,3- and α2,6-linked sialylation observed. Nine of the *O*-glycans were significantly altered between the two groups. The most significant changes were observed in core-2 structures that were mono- (^A^(Hex)2 (HexNAc)2 (Fuc)1 (NeuAc)1, *p*=0.0004) or di-sialylated (^B^(Hex)3 (HexNAc)3 (NeuAc)2, *p*=0.0005).

### Effects of GCNT3 depletion on the tumor cell glycosphingolipid synthesis

**Fig. 9:**
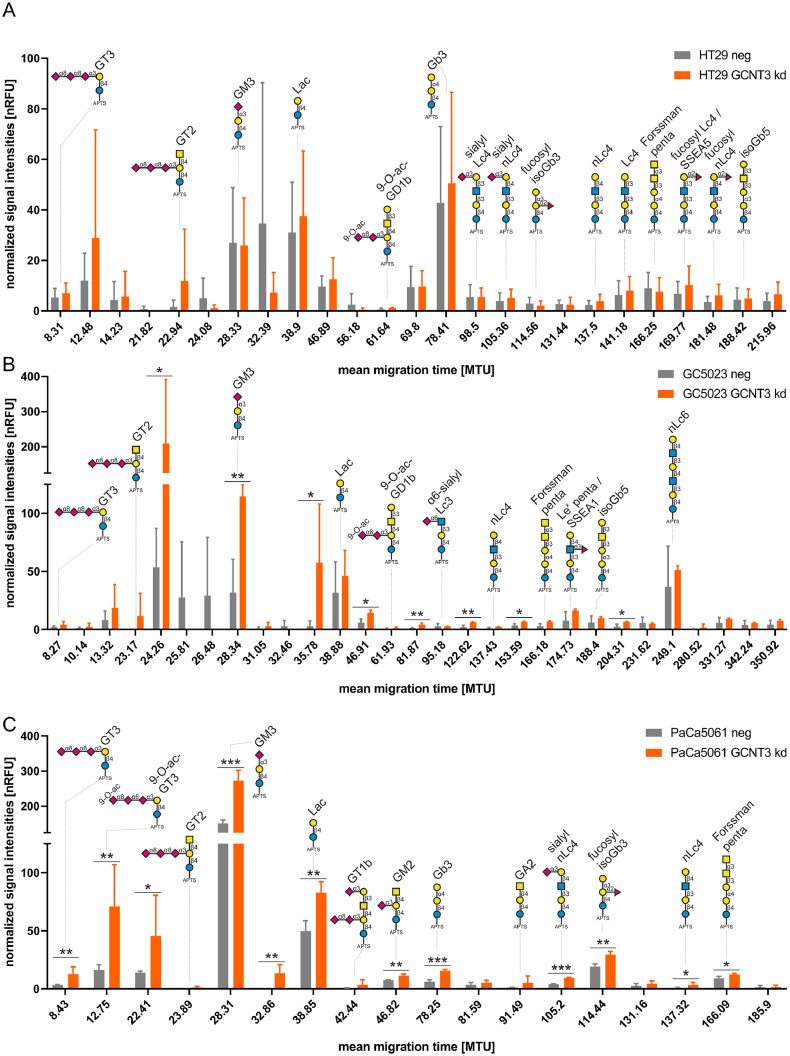
Effects of GCNT3 depletion on glycosphingolipid synthesis. xCGE-LIF analysis of glycosphingolipid-derived glycans in whole cell glycosphingolipid extracts after conventional cultivation of control (neg, grey) or *GCNT3* knockdown (kd, orange) HT29 (A), GC5023 (B) or PaCa5061 cells (C). Cells were harvested at similar confluence (∼80 %) and equal cell numbers of neg and kd were used for glycolipid extraction. Glycans that could not be annotated based on migration time matching to our in-house database are depicted. Note different y-axis scales in A and B/C. Bar charts represent mean ± SD of n = 3. nRFU = normalized relative fluorescence units, MTU = migration time units, *p < 0.05; **p < 0.001; ***p < 0.0001. Symbol key: blue circle: glucose, yellow circle: galactose, blue square: *N*-acetylglucosamine, yellow square: *N*-acetylgalactosamine, purple diamond: *N*-acetylneuraminic acid, red triangle: fucose.

Analysis of GSL glycosylation by xCGE-LIF revealed 25, 29 and 18 different glycan peaks in HT29, GC5023 and PaCa5061 cells, respectively, of which 15, 11 and 13 different glycan structures could be assigned based on our in-house glycan database (Fig. 9A-C). This observation suggests that the diversity of GSLs is less complex in PaCa5061 cells compared to HT29 and GC5023 cells, as also observed on protein linked glycosylation. Levels of diverse GSL-glycan species were found to differ significantly between the mock-transfected and the *GCNT3* kd group in GC5023 and PaCa5061 cells. Notably, in all cases, the *GCNT3* kd by trend led to an up-regulation of the respective glycan structures. However, no significant changes were observed for the HT29 cell line (Fig. 9A). In short, the *GCNT3* kd significantly increased the levels of the ganglioside GM3 in GC5023 and PaCa5061 cells (*p*<0.01). The abundances of GT3, GT2, Lac, 9-O-ac-GD1b, α6-sialyl Lc3, nLc4, Forssman penta, Le^x^ penta/ SSEA1, isoGb5, and nLc6 remained unaltered upon *GCNT3* kd in GC5023 cells (Fig. 9B). In PaCa5061 cells, the *GCNT3* kd significantly increased the amounts of GM3, Gb3, sialyl nLc4 (all with p<0.0001), GT3, 9-O-ac-GT3, Lac, GM2, fucosyl isoGb3 (all with *p*<0.001), and of nLc4, and Forssman penta (all with p<0.05) as well as two further structures that could not be assigned so far (Fig. 9C). Levels of GT2, GT1b, and GA2 did not change.

## Discussion

Little is known about the functional role of GCNT3 in cancer metastasis. A current PubMed search (September 2024) with the term “GCNT3 metastasis” reveals 8 results only. Among the few studies available, GCNT3 has been linked to both tumor progression-promoting as well as -inhibiting roles depending on the tumor type [[9], [10], [11], [12]], suggesting the need for further research. A comprehensive understanding of GI cancer glycomics remains elusive, largely due to the variation in sample types and analytical methods employed. While recent advancements in analytical techniques have shown promise, challenges persist. Recently, Wang et al. conducted a study using mass spectrometry to compare the glycosylation profiles of *N*-glycans, *O*-glycans, and glycosphingolipid glycans across 22 colorectal cancer cell lines [22]. Their analysis revealed a high abundance of (sialyl)Lewis antigens in colon-like cell lines, whereas undifferentiated cell lines displayed elevated levels of H blood group antigens and α2-3/6 sialylation. The authors suggest that the overexpression of (sialyl)Lewis antigens, particularly sLeA, could serve as promising diagnostic biomarkers and therapeutic targets for well-differentiated colorectal cancer cells.

We previously reported that GCNT3 kd in human colon cancer HT29 cells leads to reduced sLeA expression and static E-selectin binding, presumably decreasing metastatic competence, while sLeX was enhanced [8]. These findings could be fully reproduced here and expanded to human gastric cancer GC5023 cells showing a similar behavior. Thus, sLeA but not sLeX strongly appears to be the ligand for E-selectin under static conditions in these cell lines. This finding supports previous observations with the same cells, where an *O*-GalNAc-glycosylation inhibitor (GalNAc-α-*O*-benzyl, reducing the available amount of the presumed substrate of GCNT3) abrogated both sLeA synthesis and static E-selectin binding. Likewise, treatment of GC5023 cells with pronase strongly reduced sLeA expression and E-selectin binding, but left sLeX levels relatively unchanged [13]. In human pancreatic cancer PaCa5061 cells, however, neither sLeA/X nor static E-selectin binding were significantly altered after GCNT3 depletion here. In our former study, static E-selectin binding by PaCa5061 cells was closely linked to sLeX expression, and both were again abrogated in presence of GalNAc-α-*O*-benzyl [13]. These findings collectively suggest that sLeX-carrying *O*-GalNAc glycans other than GCNT3 products might mediate E-selectin binding specifically in PaCa5061 cells.

Taking all three tested cell lines together, our data demonstrate that GCNT3 products are possible scaffolds of sLeA, but not sLeX, mediating high affinity, static E-selectin binding in a part of human GI adenocarcinoma cells. This finding is in contrast with previous studies suggesting core 2 sLeX as a high affinity selectin ligand [23,24].

However, the emerging picture is complicated by our previous observation that sLeA/X presentation and static E-selectin binding were pronase-insensitive in case of HT29 and PaCa5061 cells (while being sensitive to GalNAc-α-*O*-benzyl [13]). Therefore, we previously assumed that GalNAc-α-*O*-benzyl might have indirectly affected the synthesis of sLeA/X-decorated glycolipids as potential E-selectin binding sites [25]. As we observed a significant loss of GM3 gangliosides after treatment with GalNAc-α-*O*-benzyl [13], we also analyzed glycolipid changes upon GCNT3 kd in the present study. However, the loss of sLeA levels and E-selectin binding after GCNT3 kd in HT29 cells was not accompanied by any significant changes in the glycolipid composition. Vice versa, PaCa5061 cells, whose sLeA/X levels and E-selectin binding capacity remained unaltered upon GCNT3 kd, showed several significant changes in glycolipids, all of which increased. Therefore, the lack of effect of pronase on sLeA/X and E-selectin binding of HT29 and PaCa5061 cells in our former study [13] could also be explained by the known resistance of mucins to protease mixtures like pronase due to their heavy mucin-type glycosylation [26]. However, the remarkable loss of larger protein bands in Coomassie stains of HT29 cell extracts after pronase treatment strongly argues against this hypothesis [16].

The common increase in several glycolipids after GCNT3 kd in PaCa5061 cells might be due to enhanced substrate availability due to the constricted *O*-glycan maturation. It has been shown previously that manipulating gene expression of glycosyltransferase from one glycan class can affect the synthesis of other glycan classes. For example, Alam et al., demonstrated that by mutating B3GNT5, the depletion of (neo-) lacto series GSLs consequently affects α2-6 sialylation on *N*-glycoproteins, not only at the cellular level, but also through the silencing of the corresponding ST6GAL1 gene [27]. However, these increased amounts of glycolipids on PaCa5061 cells could not be functionally related to sLeA/X presentation or E-selectin binding in the present study.

This study revealed cell line-specific overall glycosylation patterns and glycosylation changes upon GCNT3 depletion in GI adenocarcinomas. Regarding *N*-glycans, the sLeA/X epitope was found at low abundance in the HT29 cells, and did not vary much between the two conditions. Instead, oligomannose glycans were the most abundant, with sialylated glycans (in both α2,3 and α2,6 linkages) being the second most common. The depletion of GCNT3 did not drastically affect the overall profile of *N*-glycans, but significant changes were observed in the abundance of specific oligomannose and sialylated structures, particularly those carrying bisecting GlcNAc residues, which are known to influence the branching and complexity of *N*-glycans. In GC5023 cells, the *N*-glycans featured a diverse array of structures, with a predominance of oligomannose types and sialylated structures. Notably, GCNT3 depletion led to an increase in bisecting GlcNAc residues, particularly in hybrid and mono/di-sialylated *N*-glycans. This change in glycosylation patterns is noteworthy, because bisecting GlcNAc structures are known to inhibit further elongation of *N*-glycan chains. The PaCa5061 cell line, which exhibited a unique profile due to the expression of LacdiNAc motifs, also displayed increased sialylation upon GCNT3 depletion.

The *O*-glycan profiles of the three cell lines revealed critical changes in LeA/X antigen and sLeA/X antigen expression following GCNT3 depletion. The HT29 cell line displayed *O*-glycan structures with a significant presence of Lewis and sialyl-Lewis antigens. The most abundant *O*-glycan structure in HT29 cells, a core-2 di-sialylated glycan, showed a notable reduction after GCNT3 depletion while core-1 structures increased. Additionally, sLeA antigens characterized by their chromatographic elution pattern [28] and negative mode fragmentation [21] were found to be significantly reduced, while an increase of the sLeX carrying isomers was observed. In GC5023 cells, more than half of the *O*-glycan structures were altered by GCNT3 depletion. Interestingly, although overall sialylation was maintained, changes in *O*-glycan core branching were observed. GC5023 cells showed decreased expression of sLeA antigens in both linear and core-2 type scaffolds. PaCa5061 cells exhibited several *O*-glycan structures, all of which were sialylated. Following GCNT3 depletion, core-2 structures were significantly altered, with mono- and di-sialylated structures being affected. Interestingly, of the six *O*-glycan structures containing the sLeA/X antigen, only two were found to be altered, both being isomers of the same glycan composition.

GCNT3 depletion also affected GSL synthesis in GC5023 and PaCa5061 cells, though no significant changes were observed in HT29 cells. In particular, several GSL-glycan species, including the ganglioside GM3, which is often associated with cell proliferation and differentiation, were up-regulated upon GCNT3 depletion. Other GSLs, including Gb3 and sialylated GSLs, were also upregulated, suggesting that GCNT3 affects both protein-linked and lipid-linked glycosylation pathways. The upregulation of specific GSLs in GCNT3-depleted cells might reflect compensatory mechanisms or alterations in cell surface dynamics that could influence tumor cell behavior.

Interestingly, the ∼50 % reduction of sLeA levels after GCNT3 kd in HT29 cells sufficiently reduced their ability to dynamically adhere on E-selectin (under flow conditions) but not on HUVECs, although HT29 cells are known to adhere on HUVECs in a strongly E-selectin-dependent manner [16]. This discrepancy might be due to cytokine-induced changes in the microtopology and avidity of the contact surfaces in the more physiological flow adhesion assay on HUVECs [29] which are irrelevant in the adhesion assay on E-selectin. On the contrary, ∼90 % reductions in sLeA levels on HT29 cells (upon neuraminidase and GalNAc-α-*O*-benzyl treatment) previously led to reduced adhesions on both E-selectin [13] and HUVECs [16]. Hence, one might hypothesize that the cytokine-related side effects on the HUVEC surface mainly affect adhesion when there is still a clear residual expression of sLeA. However, this hypothesis only seems to apply to HT29 cells, as in case of GC5023 cells the ∼30 % reduction in sLeA levels after GCNT3 kd did not change dynamic adhesion on E-selectin but increased the number of more loose adhesion (rolling instead of firm) on HUVECs. This switch in the predominant adhesive quality was also observed with GC5023 cells upon neuraminidase and GalNAc-α-*O*-benzyl treatment [16] and could be interpreted as an indication of impaired adhesion capacity. Here, the effects of the GCTN3 kd on dynamic adhesion seem to be even more subtle and not detectable on a pure E-selectin coating. To explain why a reduction in adhesion ability to E-selectin was only observed in HT29 cells, it should also be considered that these were the cells with the lowest overall expression of sLeA/X among the cell lines tested. Thus, falling below a critically low sLeA level might disrupt the dynamic tumor-E-selectin-interaction.

The only phenotype that was common among all tested cell lines was the reduced migratory behavior upon GCNT3 depletion while effects on tumor cell proliferation (2D) and colony forming capacity (3D) varied inconsistently between the cell lines. Our findings are in line with a previous study showing that miR-BART1-5p, directly targeting GCNT3, as well as GCNT3 knockdown inhibited migration in EBV-associated gastric cancer [30]. Likewise, down-regulation of GCNT3 by miR-302b-3p impaired migration of non-small cell lung cancer cells [31]. Furthermore, silencing and functional inhibition of GCNT3 were found to suppress migration of melanoma cells [32]. However, in the context of castration-resistant prostate cancer, GCNT3 products appear to play an opposing role since migration and epithelial-to-mesenchymal transition were induced after GCNT3 depletion [33].

The downstream effects of GCNT3 depletion that might account for the impaired migratory behavior remain to be determined. Future studies should investigate which pro-migratory molecules at the cell surface are glycosylated by GCNT3 and whether their half-life or functionality depend on GCNT3 activity. One further limitation is the incomplete depletion of GCNT3 using shRNA; however, complete losses of gene expression, as can be achieved e.g. with a CRISPR/ Cas9 approach, ultimately create unnatural states that are not taken up by metastatic cells in particular. Instead, the ability to swiftly adapt expression levels across a wide range (in terms of cellular plasticity) is pathophysiologically advantageous for metastasizing cells; in this regard, gene expression is most likely not completely switched off in most cases. In our opinion, this situation is better simulated by the chosen shRNA approach.

Future *in vitro* studies on GCNT3 should consider our observation that tumor cells might increase GCNT3 levels across the first passages after revitalisation, suggesting that the glycosylation machinery of tumor cells needs some time to adapt to culture conditions after thawing. This observation is particularly important for xeno-transplantation studies, where the tumor cells are usually freshly thawed, rapidly expanded and transplanted into mice within the first two to three passages. Since initial growth in new environments most likely depends on tumor-host interactions and thus also on the composition of the tumor cell glycocalyx, slightly different passage numbers before transplantation could have drastic effects on the results of the transplantation model. On the other hand, we observed that the lentiviral transduction and puromycin selection procedure per se does not alter GCNT3 levels. HT29 and GC5023 cells would be the primary candidates for xenograft experiments as they represent the high GCNT3 expression levels observed in GI cancer patients and show concordant changes in sLeA and sLeX levels upon GCNT3 kd.

Summarized, this study demonstrates that sLeA can be carried by GCNT3 products and as such mediate static E-selectin binding in two of the three tested GI adenocarcinoma cells, HT29 and GC5023. Further structures carrying sLeX must exist that mediate E-selectin binding in the tested pancreatic cancer cell line PaCa5061. Effects on sLeA levels and static E-selectin binding are not necessarily reflected in dynamic adhesion assays on E-selectin or endothelial cells. GCNT3 products inconsistently affect tumor cell proliferation and colony formation capacity but are common pro-migratory structures in GI adenocarcinoma.

## Availability of data and materials

This work solely includes data generated in the course of the study and all data on which the conclusions rely are made available in the manuscript. Materials are available from the corresponding author on reasonable request.

## List of abbreviations

CAM, cell adhesion molecule; CTC, circulating tumor cell; EC, endothelial cell; E-Sel, recombinant human E-selectin; HUVEC, human umbilical vein endothelial cell; sLeA, sialyl-Lewis A; sLeX, sialyl-Lewis X; TC, tumor cell.

## CRediT authorship contribution statement

**Lisa Staffeldt:** Writing – original draft, Validation, Methodology, Investigation, Formal analysis. **Hanna Maar:** Methodology, Investigation, Formal analysis. **Julia Beimdiek:** Methodology, Investigation. **Samuel Chambers:** Methodology, Investigation. **Kristoffer Riecken:** Writing – review & editing, Resources. **Mark von Itzstein:** Resources. **Falk F.R. Buettner:** Writing – review & editing, Supervision, Methodology, Investigation, Funding acquisition, Conceptualization. **Arun Everest-Dass:** Writing – review & editing, Writing – original draft, Supervision, Methodology, Investigation, Conceptualization. **Tobias Lange:** Writing – review & editing, Writing – original draft, Supervision, Resources, Funding acquisition, Conceptualization.

## Declaration of competing interest

The authors declare that they have no known competing financial interests or personal relationships that could have appeared to influence the work reported in this paper.

## Appendix Supplementary materials

Image, application 1
