# Corrigendum to “Depletion of ß1,6-N-acetylglucosaminyltransferase reduces E-selectin binding capacity and migratory potential of human gastrointestinal adenocarcinoma cells” [Neoplasia 59 (2024) 10183]

**DOI:** 10.1016/j.neo.2025.101124

**Published:** 2025-01-17

**Authors:** Lisa Staffeldt, Hanna Maar, Julia Beimdiek, Samuel Chambers, Kristoffer Riecken, Mark von Itzstein, Falk FR Buettner, Arun Everest-Dass, Tobias Lange

**Affiliations:** a Institute of Anatomy and Experimental Morphology, University Cancer Center Hamburg, University Medical Center Hamburg-Eppendorf, 20241, Hamburg, Germany; b Institute of Anatomy I, Jena University Hospital, 07743, Jena, Germany; c Comprehensive Cancer Center Central Germany (CCCG); d Institute of Clinical Biochemistry, Hannover Medical School, 30625, Hannover, Germany; e Proteomics, Institute of Theoretical Medicine, Faculty of Medicine, University of Augsburg, Augsburg, Germany; f Institute for Glycomics, Griffith University, Gold Coast Campus, Gold Coast, QLD4222, Australia; g Research Department Cell and Gene Therapy, Department of Stem Cell Transplantation, University Medical Center Hamburg-Eppendorf, 20246, Hamburg

The authors regret that a correction of the affiliations is necessary. In the original paper, Dr. Falk FR Buettner was incorrectly affiliated to ^a^Institute of Anatomy and Experimental Morphology, University Cancer Center Hamburg, University Medical Center Hamburg-Eppendorf, 20241, Hamburg, Germany.

The correct affiliation of Dr. Falk FR Buettner is Institute of Clinical Biochemistry, Hannover Medical School, 30625, Hannover, Germany and Proteomics, Institute of Theoretical Medicine, Faculty of Medicine, University of Augsburg, Augsburg, Germany

Also Dr. Arun Everest-Dass in the original paper was incorrectly tagged to Institute of Anatomy and Experimental Morphology, University Cancer Center Hamburg, University Medical Center Hamburg-Eppendorf, 20241, Hamburg, Germany. But the correct affiliation of Dr. Arun Everest-Dass is Institute for Glycomics, Griffith University, Gold Coast Campus, Gold Coast, QLD4222, Australia

The authors would like to apologise for any inconvenience caused.

